# Ensemble Machine Learning Model to Predict SARS-CoV-2 T-Cell Epitopes as Potential Vaccine Targets

**DOI:** 10.3390/diagnostics11111990

**Published:** 2021-10-26

**Authors:** Syed Nisar Hussain Bukhari, Amit Jain, Ehtishamul Haq, Abolfazl Mehbodniya, Julian Webber

**Affiliations:** 1 University Institute of Computing, Chandigarh University, NH-95, Chandigarh-Ludhiana Highway, Mohali 140413, India; amit_jainci@yahoo.com; 2 Department of Biotechnology, University of Kashmir, Srinagar 190006, India; haq@uok.edu.in; 3 Department of Electronics and Communication Engineering, Kuwait College of Science and Technology, Kuwait City 13133, Kuwait; a.niya@kcst.edu.kw; 4 Graduate School of Engineering Science, Osaka University, Toyonaka, Osaka 560-8531, Japan; jwebber@ieee.org

**Keywords:** COVID-19, SARS-CoV-2, T-cell epitope, peptide-based vaccines, machine learning, random forest, ensemble learning, voting ensemble

## Abstract

An ongoing outbreak of coronavirus disease 2019 (COVID-19), caused by a single-stranded RNA virus called severe acute respiratory syndrome coronavirus 2 (SARS-CoV-2), has caused a worldwide pandemic that continues to date. Vaccination has proven to be the most effective technique, by far, for the treatment of COVID-19 and to combat the outbreak. Among all vaccine types, epitope-based peptide vaccines have received less attention and hold a large untapped potential for boosting vaccine safety and immunogenicity. Peptides used in such vaccine technology are chemically synthesized based on the amino acid sequences of antigenic proteins (T-cell epitopes) of the target pathogen. Using wet-lab experiments to identify antigenic proteins is very difficult, expensive, and time-consuming. We hereby propose an ensemble machine learning (ML) model for the prediction of T-cell epitopes (also known as immune relevant determinants or antigenic determinants) against SARS-CoV-2, utilizing physicochemical properties of amino acids. To train the model, we retrieved the experimentally determined SARS-CoV-2 T-cell epitopes from Immune Epitope Database and Analysis Resource (IEDB) repository. The model so developed achieved accuracy, AUC (Area under the ROC curve), Gini, specificity, sensitivity, F-score, and precision of 98.20%, 0.991, 0.994, 0.971, 0.982, 0.990, and 0.981, respectively, using a test set consisting of SARS-CoV-2 peptides (T-cell epitopes and non-epitopes) obtained from IEDB. The average accuracy of 97.98% was recorded in repeated 5-fold cross validation. Its comparison with 05 robust machine learning classifiers and existing T-cell epitope prediction techniques, such as NetMHC and CTLpred, suggest the proposed work as a better model. The predicted epitopes from the current model could possess a high probability to act as potential peptide vaccine candidates subjected to in vitro and in vivo scientific assessments. The model developed would help scientific community working in vaccine development save time to screen the active T-cell epitope candidates of SARS-CoV-2 against the inactive ones.

## 1. Introduction

An infection outbreak caused by a novel coronavirus has proliferated rapidly around the world. The World Health Organization (WHO) designated the disease as COVID-19 [1,2]. The pathogen was named SARS-CoV-2 by the Coronaviridae Study Group (CSG) [3]. The pathogen has resulted in 225,488,491 COVID-19 cases and 4,644,376 deaths worldwide as of September 13, 2021, posing a significant challenge to public health worldwide [4]. Furthermore, because SARS-CoV-2 keeps on circulating, the chances of mutations in the virus also increases. The recent delta variant with *Pango lineage* as AY.1, AY.2, AY.3, and B.1.617.2 was first identified in India in April-May 2021 [5]. The spike protein substitutions for the delta variant are T19R, (V70F*), T95I, G142D, E156-, F157-, R158G, (A222V*), (W258L*), (K417N*), L452R, T478K, D614G, P681R, and *D950N* [5]. The lineage proliferated quickly and demonstrated somewhat partial resistance to existing vaccines. The variant pushed the confirmed cases in India to 400,000 plus a day [6]. On June 15, 2021, it was declared as a variant of concern (VOC) [7,8]. According to a recent study published in the Chinese Academy of Medical Sciences, “viral loads in Delta infections are [about] 1,000 times higher” than those caused by prior SARS-CoV-2 variants [9]. In such situations (virus mutations), existing vaccines may prove to be somewhat less effective against new strains. To guard against these mutations, the only option is to either adjust the composition of the existing vaccines or produce new vaccines [10].

*Coronaviruses* (CoVs) are members of the *Coronaviridae* family of viruses. They are enveloped viruses with extremely long single-stranded RNA genomes ranging from 26 to 32 kilobases in length [11] and their structure is similar to that of known CoVs. Almost 2/3 of the 5′ genome region is constituted by *orf1ab* genes that encode *orf1ab* polyproteins. In contrast, 1/3 of 3′ is formed by genes that encode the structural proteins, i.e., *envelope-E, surface-S, nucleocapsid-N, and membrane-M* [12]. In a study conducted by Lineburg and colleagues [13], it has been found that SARS-CoV-2 consists of 26 viral proteins. Among them are some surface proteins, such as *spike-S* protein, and others are more conserved and internal, i.e., *nucleocapsid-N* protein. The sequence conservation of non-surface proteins qualifies them as prime vaccine targets for cytotoxic CD8+ T-cell activation.

The SARS-CoV-2 infection triggers both innate and adaptive immune responses [14]. Viruses are generally detected and processed by antigen-presenting cells. The CD4+ T cells primarily differentiate into effector cells that release *cytokines* and *chemokines* after T-cell activation; cytotoxic CD8+ T cells are essential players in the immune response to viral infections because they directly participate in viral clearance [15]. T cells have been shown to be the target structural proteins of *coronaviruses* and are implicated in immunopathological lung damage in SARS-CoV and MERS-CoV [16,17]. Identification of viral T-cell epitopes on human leukocyte antigens (HLA) is required to characterize T-cell immunity and develop vaccines and immunotherapies [18,19]. T-cell activation occurs when SARS-CoV-2 peptides are recognized on the infected cell surface via a HLA (human leukocyte antigen) molecule [20]. A large international effort is still underway to devise different strategies to fully contain coronavirus infections caused by SARS-CoV-2 and control the virus mutations. Thus, it becomes vital to apply an epitope-based peptide vaccine development approach that is cost-effective, safe, and takes less time compared to inactivated vaccine, live-attenuated vaccine, and viral vector vaccine approaches. To design an epitope-based peptide vaccine, it is essential to identify and select T-cell epitopes which are antigenic. With ML techniques the T-cell epitopes can be predicted with high accuracy. It would also save more physical experimental time and effort for speedy vaccine development compared to wet-lab techniques. This study proposes an ensemble machine learning model to predict T-cell epitopes of the SARS-CoV-2 virus. The predicted T-cell epitopes can act as potential vaccine targets for developing an epitope-based peptide vaccine against this pathogen.

### 1.1. Related Work

According to the literature, researchers started using machine learning methods reasonably quickly once the initial genome sequences of SARS-CoV-2 became public in early 2020 to recommend T-cell epitopes which are antigenic in nature as potential vaccine candidates against this pathogen [21]. In their study, Naz et al. [22] have reported a collection of SARS-CoV epitopes (T and B cell) from the spike and nucleocapsid protein parts by taking into consideration the scientifically proven fact that SARS-CoV and SARS-CoV-2 are genetically very similar. As per their study, the epitopes screened using existing bioinformatics tools can help experimental efforts in developing a vaccine against the SARS-CoV-2 pathogen. Grifoni et al. [23], in their study, have retrieved peptide sequences of SARS-CoV from the IEDB [24] repository because SARS-CoV-2 has high genetic similarity to SARS-CoV [22]. Later, a number of candidate B-and T-cell epitopes were identified for SARS-CoV-2 using bioinformatics-based prediction tools. The predicted epitopes could act as potential vaccine targets and help design an effective vaccine against the SARS-CoV-2 virus. In their work, Baruah et al. [25] have used immunoinformatics to identify prominent B-cell and (Cytotoxic T lymphocyte) CTL epitopes from surface glycoprotein of SARS-CoV-2. Interactions between identified CTL epitopes and their associated MHC class I supertypes were further explored using molecular dynamics simulations. From the surface glycoprotein of the virus, five (05) CTL, three (03) sequential, and five (05) discontinuous B-cell epitopes were identified. Some of the identified epitopes have been considered as suitable candidates for SARS-CoV-2 vaccine development. In their study, [22] have explored the spike protein to identify epitopes that are immunogenic for epitope-based vaccine design against SARS-CoV-2. Two portions, i.e., S1, S2 of the spike protein, were later analyzed and two vaccine constructs were prioritized with B- and T-cell epitopes. The epitopes so prioritized have been modelled using adjuvants and linkers by creating their 3D models to assess their physicochemical properties and possible interaction with HLA, ACE2, and TLR2, as well as TLR4. In their study, Crooke et al. [26] have established a computational approach for analyzing the SARS-CoV-2 proteome and identified probable B- and T-cell epitopes utilizing several open-source web tools and algorithms. After applying the defined computational approach, a total of forty-one (41) and six (6) B- and T-cell epitopes were identified. These epitopes could act as potential targets for designing the peptide-based vaccine against the SARS-CoV-2 virus. Dong et al. [27], in their study, attempted to build a multi-epitope vaccine for treatment and prevention of COVID-19 using immunoinformatics methods. The epitopes were computed by using B cells, CTLs, and (Helper T lymphocytes) HTLs of SARS-CoV-2 proteins. By combining the B-cell, CTL, and HTL epitopes with linkers, a vaccine was finally devised. The EAAAK linker was used to attach the 45-mer peptide sequence, called β -defensin and pan-HLA binding peptide (13aa), to the vaccine’s N-terminus to improve immunogenicity.

The existing methods based on machine learning, which researchers have utilized, can either predict CD8+ or CD4+ T-cell epitopes and are listed in Table 1.

Few techniques listed above have ‘pan’ as a suffix, which means an ability to predict the binding of HLA’s peptide for a huge collection of the alleles inside a particular HLA type, including those not present in the training dataset [37]. Few studies have also used algorithms specific to HLA-I, namely, NetCTL1.2 [39] and Net_Chop [40], where extra intracellular variables responsible for presentation of HLA antigen were integrated to improve the prediction accuracy of binding the peptide HLA. The methods NetCTL-1.2 [39] and NetChop [40] have also been utilized by few studies where extra intracellular variables have been integrated, which are responsible for presenting HLA antigen. The main aim was to improve the prediction accuracy of peptide HLA binding. It is essential to mention here that almost all modern T-cell epitope prediction systems use artificial neural networks (ANNs). A few early ones (such as RANKPEP [41] and CTLPred [42]) used a different ML approach, support vector machines (SVMs). Meyers et al. [43], in their study, have identified T-cell epitopes of SARS-CoV-2 using immunoinformatics based methods by taking onto account T cell epitopes from envelope, membrane, and spike portions of the pathogen having maximum potential HLA binding. Other factors used for selecting T-cell epitopes were HLA diversity and circulating virus coverages as well as “minimum cross-reactivity with self”. To identify CD8 T-cell epitopes in SARS-CoV-2 proteome which are mutationally constrained, Nathan et al. [44], in their study, have used a structure-based analysis of network and assessments of class I HLA sequences stability. These findings identify mutationally restricted areas and epitopes of the SARS-CoV-2 proteome which are immunogenic and that could be used to develop a global T cell-based vaccination against new variants and SARS-like coronaviruses.

### 1.2. Motivations

There are four main motivations behind this study:

1. There are numerous drawbacks to using whole-organism vaccines, particularly in immunocompromised patients [45,46]. Epitope-based peptide vaccines can be utilized to overcome the issues associated with multicomponent and heterogeneous vaccines. They can act as powerful alternatives to conventional vaccines due to their low production cost, and less reactogenic and allergenic responses.
2. The majority of the existing methods, as mentioned in Section 2, utilize ANNs [21] and few others utilize only SVM. However, ANNs are hardware dependent since they demand parallel processing power, depending on their structure [47]. Moreover, instead of relying on predictions by single classifiers, we can combine predictions from more powerful classifiers and combine them using an ensembling approach. Performance of the ensemble model, in terms of accuracy, is high and it is also considered a robust model [48,49].
3. Furthermore, the majority of the methods described in Section 1.1 estimate peptide binding capacity. For these methods, it remains a problem to predict directly whether a particular peptide is a SARS-CoV-2 epitope or not. One method, namely, CTLpred [42], predicts directly, but the length of the peptide sequence is limited to 9-mers only. Therefore, a direct approach to T-cell epitope prediction has been proposed here, which resolves the first problem. The proposed ensemble model can predict epitopes having variable length (length > 9-mers), fixing the second problem associated with the existing methods.
4. Because the SARS-CoV-2 virus is widely circulating in the community, the virus’s ability to mutate further is increasing. The recently discovered delta variant (B.1.617.2) is causing widespread problems [50]. Delta appears to be approximately 60% more transmissible than alpha (B.1.1.7) [6,7,9]. Existing vaccines may prove to be somewhat less effective against new variants. To protect against these variants, either the composition of existing vaccines has to be modified or a new vaccine is to be developed [10]. Time being the critical factor, an epitope-based peptide vaccine can be a great alternative, relying on their low costs, reduced time to production, being safe, and having potential for increasing immunogenicity and cross reactivity.

### 1.3. Contributions

The main contributions of this study are:

- To develop an ensemble machine learning (ML) model for SARS-CoV-2 T-cell epitope prediction. The predicted epitopes of SARS-CoV-2 would act as potential vaccine candidates against this pathogen.
- The main focus is on accuracy, which is considered an essential criterion for epitope prediction. Moreover, other metrics such as AUC, sensitivity, precision, Gini, specificity, and F-score have been used for model evaluation.
- To carry out the comparative analysis of the proposed ensemble model with various existing prediction models, namely, support vector machine, random forest, neural network, decision tree, and adaBoost.
- To compare the proposed ensemble model with existing benchmark techniques using blind dataset.
- To assess the effectiveness of the proposed ensemble classification model using a technique called repeated 5-fold cross validation.
- To our knowledge, this is the first study to propose an ensemble ML model to predict T-cell epitopes of SARS-CoV-2 virus as potential vaccine targets for designing an epitope-based peptide vaccine.

## 2. Materials and Methods

### 2.1. Retreival of SARS-CoV-2 Peptide Sequences

To develop an effective and viable epitope-based peptide vaccine against the various strains of SARS-CoV-2, it is essential to select the antigenic T-cell epitopes. The experimentally determined linear peptide sequences (T-cell epitopes and non-epitopes) were retrieved from IEDB [24]. IEDB is a freely available resource funded by the National Institute of Allergy and Infectious Diseases (NIAID), with its headquarters at North Bethesda, MD, USA. It catalogs experimental data on antibody and T-cell epitopes studied in humans, non-human primates, and other animal species in the context of infectious disease, allergy, autoimmunity and transplantation. The data was retrieved in comma-separated values (CSV) format in two files, with one file containing epitope and another non-epitope sequences. Retrieved data consists of 10485 peptide sequences, of which 1744 are T-cell epitopes and 8741 are non-epitopes. Because this is a binary classification problem, we included “Class” as a target variable in both CSV files, with values of 1 for epitope sequences and 0 for non-epitope sequences.

### 2.2. Proposed Methodology

**Figure 1:**
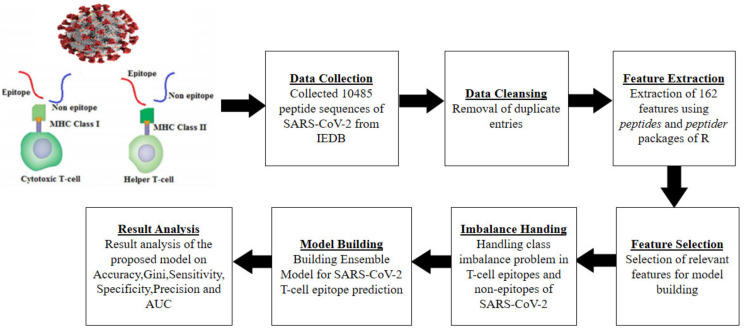
Proposed methodology.

The proposed methodology for building the proposed ensemble model is depicted in Figure 1 and is explained through the following steps.

#### 2.2.1. Data Cleansing and Feature Extraction

After obtaining the peptide sequences in CSV file format, the next step was to extract features. We extracted the features (physicochemical properties [51,52]) from two different CSV files containing the peptide sequences of the SARS-CoV-2 virus using peptides [51] and peptider [52] packages of R [53] programming language. Few duplicate entries were eliminated before performing feature extraction. For each sequence in CSV files, the feature extraction generated a high dimensional dataset consisting of 162 features. These two datasets corresponding to two CSV files were merged later into one CSV file. The physicochemical properties used in the current study are illustrated in Table 2. The dataset generated after feature extraction is displayed in Table 3.

#### 2.2.2. Feature Selection

Feature selection is a technique of selecting important and relevant features to improve the performance of an ML classifier. Features that provide irrelevant information and those that are less important are eliminated from the dataset. Since the dataset is high dimensional, consisting of 162 features, it is important to identify the best subset of features. In this study, the feature selection process was carried out using the Boruta() [54] function in R. Boruta() is a wrapper function that finds the important features by considering the values of minImp, maxImp, meanImp, medianImp, and normHits [54]. The input arguments to Boruta() is the dataset of 162 features and the outcome variable (Class). After its execution, it returned only 20 important attributes out of 162. Only these 20 attributes were used for building the proposed model. Table 4 list these important features. The importance of features is listed in decreasing order of rank with rank 1 as highly important.

#### 2.2.3. Class Imbalance Handling

One of the major issues in ML is the class imbalance problem where the primary class of interest is frequently uncommon and causes a bias in the model. The peptide sequences dataset obtained from IEDB [24] was found to be highly imbalanced. The number of epitopes were 1744 (minority class) and non-epitopes 8741 (majority class). The number of non-epitopes is nearly five times more than the number of epitopes. To mitigate this problem, we divided the non-epitopes dataset into five data frames. Subsequently, we added the epitope dataset copy to all of the data frames. At this point, all of the five (5) frames contain about an equal number of epitopes and non-epitope data instances. So, these five (5) data frames are now balanced and different.

#### 2.2.4. Preparing Blind Dataset for Comparative Analysis

Before fixing the class imbalance, we extracted five (05) peptide sequences from the epitopes and non-epitopes classes. We reserved them for comparing the proposed ensemble model to the most commonly used existing techniques for T-cell epitope prediction (NetMHC and CTLpred prediction servers in this case). These ten (10) peptide sequences acted as a blind dataset because these sequences are neither part of the training set nor testing set, and were hence unseen to the developed model.

#### 2.2.5. Model Building Using Voting Ensemble

Ensemble learning (EL) enhances classification accuracy by integrating several basic classifiers in series [55,56], and in the current study we have proposed a voting-based ensemble model. Research conducted by Bukhari et al. [57] on Zika virus epitope prediction for epitope-based peptide vaccine design, demonstrated that building a voting-based ensemble model for epitope prediction is considered a reliable and effective technique because epitope prediction is a delicate and sensitive task. In voting ensemble, the base classifiers vote for a new data instance and based on the majority of the votes, a class label is returned. An ensemble model proposed in the current study is based on five base classifiers, i.e., five random forest classifiers, and combines the predictions from all these five base classifiers. For a given peptide, each base classifier will predict its label, i.e., epitope or non-epitope (vote by a given base classifier). Since it is a voting ensemble and the predictions for each label are summed and the label with the majority vote is predicted, the ensemble model can be developed either using homogeneous or heterogeneous base classifiers. We have used homogeneous approach because our main intention is to use the most robust base classifier among all available machine learning classifiers. The random forest classifier is considered as the most robust and powerful classifier among all. Second motivation for building the ensemble model based on homogenous classifiers is whether we want to split the dataset into multiple different data frames or use the entire dataset for training the base classifiers. Keeping this mind, we can create an ensemble model by training homogeneous base classifier using a particular subset of the original dataset, i.e., data frame or training the heterogeneous base classifiers using the entire training set. Training homogeneous base classifiers on subsets takes less time compared to training heterogeneous base classifiers on the entire training set. The model proposed in the manuscript is based on the first approach where the training set was divided into different splits to improve model performance, ideally achieving better performance than any single model used in the ensemble. The random forest classifier has been used as a base learner on all the splits because its performance is better than other classifiers.

**Figure 2:**
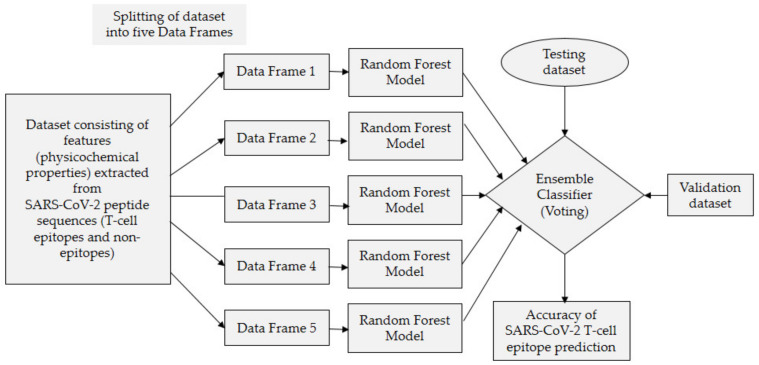
Proposed ensemble model.

Now, we have five different and balanced datasets. Figure 2 depicts the ensemble learning technique used in the current study to build the proposed ensemble model. To predict whether a particular peptide is an epitope or non-epitope, each base classifier will predict the label of that peptide, i.e., epitope or non-epitope. Then predictions for each label are summed up and the label with the majority vote is predicted. Suppose the given peptide is “SFYVYHK” and the predictions for the label “epitope” is represented by 1 and for “non-epitope” by 0. Suppose the five random forest base classifiers used are represented by RF1, RF2, RF3, RF4, and RF5 and their predictions for the given peptide are 1, 1, 0, 1, and 0. As can be seen, there are three votes for epitope, i.e., 1, and two vote for non-epitope, i.e., 0. The majority vote is for label epitope (three 1′s) and hence the peptide is classified as an epitope.

As shown in Figure 2, we trained all random forest base classifiers using 80% of the total data and combined all of them using an EL technique. Following that, a test set that consists of 20% of tuples from each frame was used to evaluate the proposed model’s performance.

#### 2.2.6. Random Forest as Base Classifier

A random forest (RF) is a supervised algorithm and is based on an ensembling technique where the base model is a decision tree. Results of the proposed model based on random forest base classifier are far better when compared to other existing classification models. The performance of a model can be improved by tuning its parameter through a process called parameter tuning. Table 5 lists various models along with their methods, tuned parameters and corresponding packages for the comparison purpose, i.e., to compare the proposed ensemble model with these standard existing prediction models. The model implementation was made in R programming language under the GNU-GPL license.

In R, the package randomForest contains a function, randomForest(), which returns a random forest classifier object. We performed the parameter tuning of “mtry” and “ntree” among its various parameters to improve its performance. The parameter “mtry” indicates the number of randomly sampled features at each split, whereas the number of trees is represented by “ntree” parameter. The random forest model used in this study achieved better performance at values 2 and 500 for “mtry” and “ntree”, respectively. The RF method used in the current study is given as: randomForest (formula, train Dataset, mtry = 2, ntree = 500). Its formula, as shown in Equation (1), shows the target “Class” and its corresponding 20 important features for model training.

Class∼f (F1, F2, F4, F6_2, F8_5, F8_19, F8_34, F9_4, F9_6, F9_29, F9_38, F10_2, F10_7, F11_5, F12_5, F12_7, F13_4, F14_9, F15_3, F15_4) (1)

As stated earlier, RF is an ensemble of decision trees. Each tree votes and a class with majority vote is returned [58,59]. The size and depth of the decision trees used directly impact the performance of the random forest. Let n be the number of data instances and d the tree depth, then the space and time complexity of the RF model is O(ntree *mtry *d *n) and O(n*d), respectively. As a result, random forest is dependent on the size and depth of the decision tree utilized [60].

#### 2.2.7. Predictions by the Proposed Ensemble Model

The test dataset was used to evaluate the prediction accuracy of the proposed model. The evaluation is performed on the basis of votes of five random forest base models, and class label is predicted based on the majority vote system of these five base models. Now the proposed model can be used for the prediction of any given SARS-CoV-2 peptide sequence. The output will be a class label, i.e., either epitope or non-epitope. When tested using a testing dataset, the model accurately predicted the testing tuples. The results are accurate and reliable because prediction is conducted through the voting mechanism of five base classifiers.

## 3. Model Evaluation

The model evaluation is the technique of assessing the performance of a model based on a variety of parameters. T-cell epitope prediction is a binary classification problem with four possible outcomes.

- True positive (TP): This means that the actual class is positive and accurately classed as such.
- True negative (TN): This means that the actual class is negative and accurately classed as such.
- False positive (FP): This means that the actual class is negative and inaccurately classified as positive.
- False negative (FN): This means that the actual class is positive and inaccurately classified as negative.

**Figure 3:**
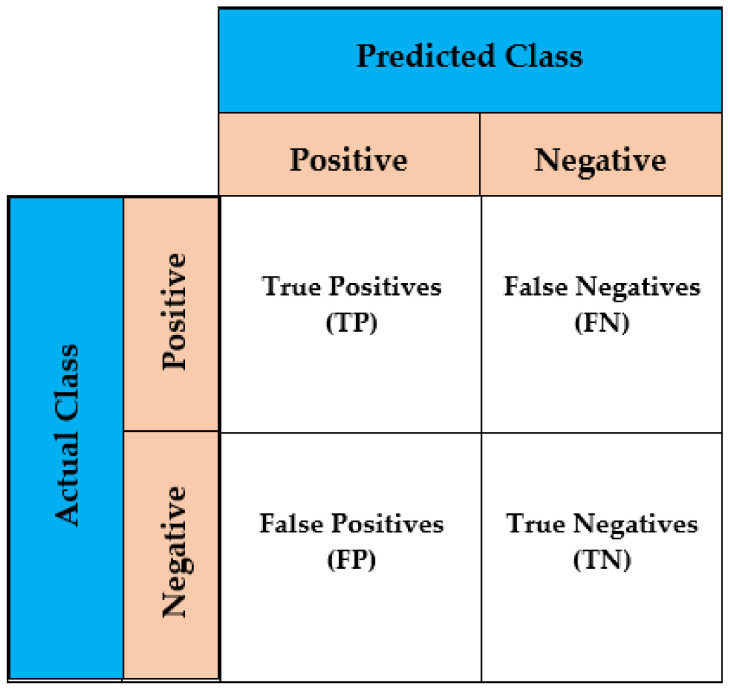
Confusion matrix.

These four outcomes are shown in a generic confusion matrix by assigning actual and predicted labels, as shown in Figure 3. A confusion matrix is a two-row, two-column table that provides the number of FPs, FNs, TPs, and TNs. This enables for more in-depth examination than a simple proportion of right classifications (accuracy). The performance evaluation metrics, such as sensitivity, specificity, Gini coefficient, precision, F-score, accuracy, and Area under ROC Curve (AUROC), are defined by using these outcomes. The robustness and consistency of the model was examined through a technique called repeated K-fold cross validation.

The quick overview of the metrics employed for model evaluation in this study is given next.

Accuracy: The model’s accuracy measures its correctness and is calculated as shown in Equation (2).

Accuracy = (TP + TN)/(TP + TN + FP + FN)(2)

Sensitivity: It is often referred as the true positive rate (TPR) or recall and is calculated as shown in Equation (3).

Sensitivity= TP/(TP + FN)(3)

Specificity: It is often referred to as true negative rate (TNR) and is calculated as shown in Equation (4).

Specificity= TN/(TN + FP)(4)

Precision: Precision is computed using Equation (5).

Precision= TP/(TP + FP) (5)

Gini coefficient: It estimates the measure of an inequality distribution in the data. It ranges from 0 to 1, with 1 denoting the perfect data inequality and 0 as perfect data equality. Suppose there are two models, X and Y, having Gini coefficients as 0.82 and 0.49, respectively, then X is more productive than Y. The Gini coefficient is computed using Equation (6).

Gini = 2 × AUC − 1 (6)

F-score: It represents the harmonic mean of recall and precision and is computed using Equation (7).

F-score = 2 × ((Recall × Precision)/(Recall + Precision)) (7)

Area under the ROC Curve: The AUROC of the model is generated by plotting it’s TPR against FPR. The AUROC is a region immediately below the ROC curve. It has a value between 0 and 1. The greater the value (near to 1), the better the model. It is computed using Equation (8).

AUROC = 1/2 × (TP/(TP + FN) + TN/(TN + FP)) (8)

**Figure 4:**
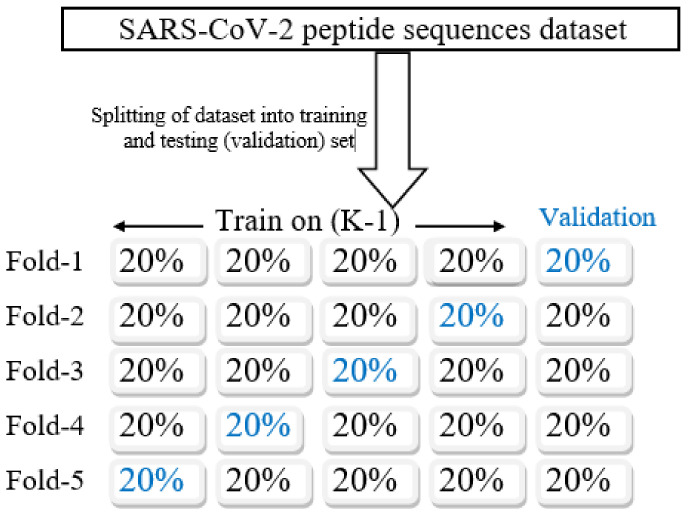
K-fold cross-validation process (K = 5).

Repeated K-fold cross validation: Evaluating the consistency and robustness of a prediction model, a prominent technique called K-fold cross validation (K-fold CV) is used [61]. It is a statistical technique used to assess the skills of any machine learning model. The dataset is partitioned into equal-sized k subsamples. In each iteration (number of iterations = k), k − 1 subsamples are used for model training; the one remaining is utilized for validation. The process is carried out so that each k subsample act as a validation set precisely once, as illustrated in Figure 4. Finally, the k iteration outcomes are averaged to achieve the model’s mean accuracy. The difference in terms of performance between two runs in K-fold CV is called noise and it is certain for a noise to be there in case of K-fold CV. The remedy to reduce the noise is to repeat the K-fold CV “n” number of times and record the mean accuracy across all the folds and repeats. This process is termed as repeated k-fold cross validation (R K-fold CV).

## 4. Results

The results achieved by the proposed ensemble model and individual classifiers, i.e., standard exiting prediction models on a dataset consisting of 20 features, are discussed in this section. The comparison results of the proposed ensemble model with individual classifier (shown in Table 6) based on performance metrics used (as discussed in Section 3) are provided here. Moreover, repeated K-fold cross-validation results are discussed to check how reliable the model is. Finally, a comparison with the two most widely used techniques for T-cell epitope prediction, i.e., NetMHC and CTLpred, is provided to demonstrate that the proposed ensemble model outperforms the existing methods.

### 4.1. Result Analysis of Comparing The Proposed Model with Existing Prediction Models

The metrics used for evaluating the performance of any classification model are the accuracy, AUC, Gini, specificity, sensitivity, F-score, and precision. Performance results of the proposed ensemble model and standard exiting prediction models using the test dataset are illustrated in Table 6. The proposed ensemble model achieved accuracy, AUC, Gini, specificity, sensitivity, F-score, and precision of 98.20 %, 0.991, 0.994, 0.971, 0.982, 0.990, and 0.981, respectively, as shown in Table 6, highlighted in bold.

**Figure 5:**
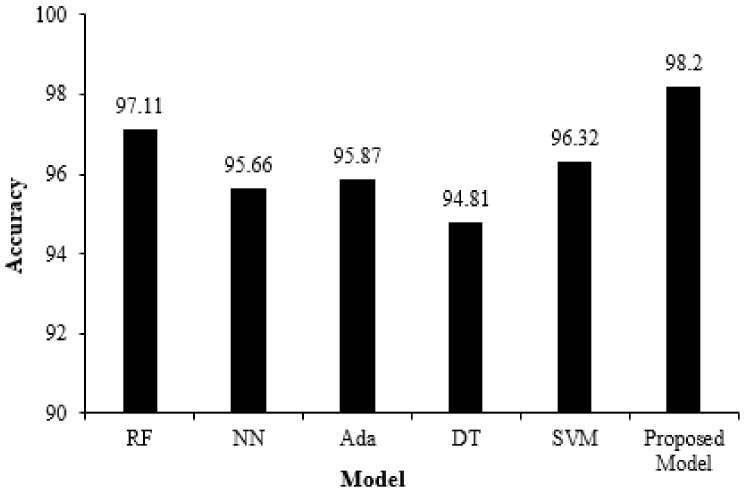
Performance comparison bar chart of individual and proposed ensemble models.

**Figure 6:**
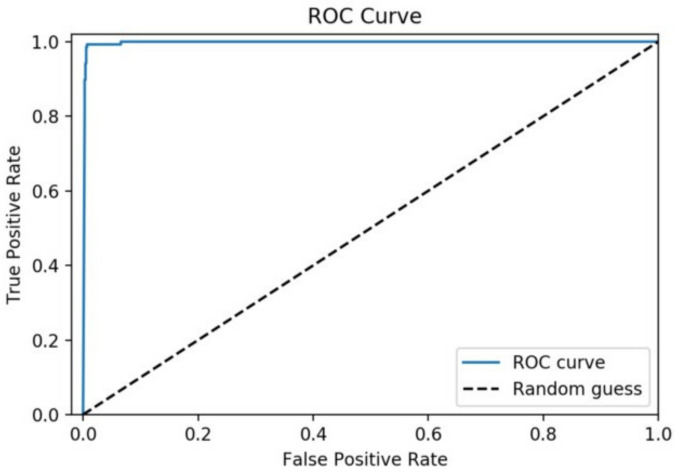
ROC curve of the proposed ensemble model.

Figure 5 depicts a performance comparison bar chart of existing models with the proposed ensemble in terms of accuracy. Figure 6 illustrates the ROC curve of the proposed model on the testing dataset and an AUROC of 0.991 has been achieved. The results indicate that the proposed ensemble model performs better than standard exiting prediction models when evaluated using the test dataset.

### 4.2. Result Analysis of Repeated K-Fold Cross Validation

**Figure 7:**
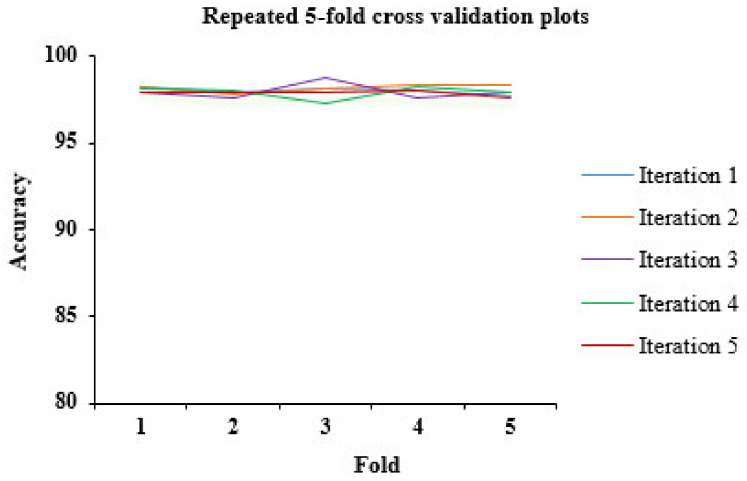
Accuracy plot of repeated K-fold cross validation (K = 5).

Another important thing to analyze is the reliability and consistency of the model; is it free from underfitting and overfitting issues? To analyze it, we carried out repeated 5-fold cross validation (5-fold CV; k = 5). The 5-fold CV process was repeated 5 times. The accuracies (in percentage) obtained iteration-wise is described in Table 7. Figure 7 depicts the accuracies plot of all iterations as recoded in a repeated 5-fold CV. The mean accuracy (mean of mean accuracies obtained per iteration) achieved through repeated 5-fold CV is 97.99%. It is visible from the results obtained through repeated 5-fold CV that the proposed ensemble model performs consistently well on all the folds iteration wise.

### 4.3. Result Ananlysis of Comparing the Proposed Model with Two Benchmark Techniques Using Blind Dataset

A separate blind dataset was used for comparative analysis in terms of accuracy of the proposed ensemble model with the two most frequently used techniques (NetMHC and CTLpred) for T-cell epitope prediction. Since the NetMHC server only estimates the binding capacity of a peptide sequence, as shown in the third column of Table 8, the proposed model is more efficient since it deterministically predicts whether a peptide is an epitope or not. The predictions by the proposed ensemble model can be seen discretely; either 1 (meaning epitope) or 0 (meaning non-epitope). This is shown in the last column of Table 8. However, CTLpred server predicts sequences in a discrete way, unlike NetMHC, but can only predict sequences of length up to 9-mers. As shown in Table 8, prediction by CTLpred for sequences having a length greater than 9-mers are represented by hyphen (-), which mean “unpredicted” as CTLpred cannot predict them. In this case, the CTLpred server could not predict epitope sequences “QLNRALTGIAVEQDK”,”NFSQILPDPSKPSKR” and “SQDLSVVSKT”. However, the proposed model classified them correctly as SARS-CoV-2 T-cell epitopes. Similarly, non-epitope sequences “EYHLMSFPQSAPHGV” and “SLPSYAAFATA” were also not predicted by CTLpred, but the proposed model predicted them correctly as SARS-CoV-2 non-T-cell epitope. Thus, the proposed model correctly classifies peptide sequences having length greater than 9-mers.

As demonstrated in Table 8, the proposed ensemble model’s prediction outcomes in terms of prediction accuracy are outstanding (100 percent in this case) since it correctly classifies all of the peptide sequences in the blind dataset. The comparison results clearly indicate that the performance of the proposed model is better as compared to existing techniques and hence outperforms the existing techniques.

## 5. Conclusions

The future of the current COVID-19 pandemic is unpredictable. Vaccines are an essential tool to fight against COVID-19. The epitope-based vaccines outshine all other types of vaccines due to their easy production process, low cost, and safety. In addition, the SARS-CoV-2 virus is continually mutating, with its recent variant called the delta variant [5]. Existing vaccines may prove to be less effective. To protect against these mutations, the existing vaccine composition must be changed or a new vaccine must be developed. Time and cost being the critical factor, epitope-based peptide vaccines can be a great alternative. To design an effective and viable peptide vaccine based on epitopes against this pathogen, i.e., SARS-CoV-2, it is essential to select the T-cell epitopes which are antigenic. An in silico machine learning approach is less costly and more stable than conventional wet laboratory experimental techniques for vaccine development. In this study, we proposed an ensemble machine learning model to predict SAR-CoV-2 virus T-cell epitopes. The main reason for using the ensemble approach is that it is more resistant to outliers and has a better chance of generalizing with future data. Feature extraction of peptide sequences obtained from IEDB [24] was performed based on physicochemical properties of amino acids using peptides and peptider packages of R. The resultant dataset was high dimensional with 162 features. To discard features with irrelevant information, the feature selection technique was carried out using the Boruta() [54] algorithm in R language. This is because choosing the right subset of features enables the model to train faster, reduces model complexity as well as overfitting, and model accuracy is improved. Another problem with the dataset was that it was highly imbalanced. To address this problem, the majority class dataset (non-epitopes) was divided into five data frames. A copy of the minority class dataset (epitopes) was added to each data frame, resulting in five data frames having almost equal numbers of epitopes and non-epitopes. For building the ensemble model, a robust and powerful classifier, namely, random forest, has been used. The ensemble model proposed is based on the majority vote of five random forest models. The model proposed was compared with standard exiting prediction models and achieved an accuracy, AUC, Gini, sensitivity, specificity, F-score, and precision of 98.20 %, 0.991, 0.994, 0.982, 0.971, 0.990, and 0.981, respectively, when evaluated on the test set. The results indicate that the proposed ensemble model performs better than the existing classification models. The repeated 5-fold cross validation was used to assess model consistency and reliability, and it was discovered that the proposed model’s performance is practically linear and a mean accuracy of 97.99% was recorded. Finally, the proposed model was compared with two widely used existing techniques for T-cell epitope prediction, namely, NetMHC and CTLpred, using a blind dataset. Results obtained clearly indicate that the proposed model performs much better than existing techniques.

In conclusion, it is clear that epitope-based vaccines have a tremendous potential and should be considered in the race for rapid development of protective vaccines against SARS-CoV-2. Nevertheless, it is pertinent to mention that some areas can be improved, such as exploring more properties of amino acids and using other ML classifiers. Therefore, in the future, we will focus on enhancing the robustness and accuracy of the predictive models by exploring more ML classifiers and the physicochemical properties of amino acids.

## Figures and Tables

**Table 1:** Existing methods for T-cell epitope prediction.

Sr. No	Method Name	Usage
01 02 03 04 05 06	NetMHC [28] NetMHCpan [29,30] NetCTLpan_1.1 [31] NetCTLpan_4.0 [28] HLAthena [32] MHCflurry [33]	To predict HLA I class or CD8+ T-cell epitopes
07 08 09 10 11	NetHMCII_2.3 [34] NetMHCIIpan_3.0 [35] NetMHCIIpan_4.0 [36] NeonMHC2 [37] MARIA [38]	To predict HLA II class or CD4+ T-cell epitopes

**Table 2:** Physicochemical properties used.

Feature Category	Physicochemical Property	Category Count	Notations Used
F1	Aliphatic Index	1	F1
F2	Boman Index	1	F2
F3	Insta Index	1	F3
F4	Probability of detection	1	F4
F5	Cross-covariance index	1	F5
F6	Hmoment Index	2	F6_1, F6_2
F7	Molecular Weight	2	F7_1, F7_2
F8	Peptide Charge for 45 scales	45	F8_1 to F8_45
F9	Hydrophobicity at 44 scales	44	F9_1 to F9_44
F10	Isoelectric Point for 9 pK scale	9	F10_1 to F10_9
F11	Kidera Factors	10	F11_1 to F11_10
F12	aaComp	18	F12_1 to F12_18
F13	FASGAI vectors	6	F13_1 to F13_6
F14	blosumIndices	10	F14_1 to F14_10
F15	protFP descriptors	8	F15_1 to F15_8
F16	Cruciani properties	3	F16_1 to F16_3

**Table 3:** Glimpse of the dataset.

Peptide Sequence	F1	F2	-----	F16_2	F16_3	Class
AFFGMSRIGMEVTPSGTW	43.33	0.3938	-----	−0.302	0.082	1
HLMGWDYPK	43.33	0.9477	-----	−0.091	−0.022	1
TGTLIVNSVLLFLAF	175.33	1.7473	-----	−0.284	−0.03	0
SVLLFLAFVVFLLVT	214	−3.036	-----	−0.156	−0.15	0

**Table 4:** Important features selected by Boruta.

Rank	Feature	Rank	Feature
1	F1	11	F9_38
2	F2	12	F10_2
3	F4	13	F10_7
4	F6_2	14	F11_5
5	F8_5	15	F12_5
6	F8_19	16	F12_7
7	F8_34	17	F13_4
8	F9_4	18	F14_9
9	F9_6	19	F15_3
10	F9_29	20	F15_4

**Table 5:** Classifiers used for comparison.

Model Name	Tuned Parameters	Method Name	Package Name
Neural network (NN)	size:10	nnet	nnet
Decision tree (DT)	maxsurrogate:0 and usesurrogate:0	rpart	rpart
Support vector machine (SVM)	type:svc and kernel: rbfdot	ksvm	kernlab
Random forest (RF)	ntree:500 and mtry:2	randomForest	randomForest
adaBoost (ada)	type: “discrete”, iter:50 and nu:0.5	ada	ada

**Table 6:** Results of proposed and existing prediction models on test dataset.

Model	Accuracy (%)	AUC	Gini	Sensitivity	Specificity	F-Score	Precision
Neural Network	95.66	0.981	0.980	0.959	0.971	0.910	0.929
Decision Tree	94.81	0.978	0.929	0.979	0.959	0.979	0.939
SVM	96.32	0.982	0.932	0.981	0.946	0.957	0.948
RandomForest	97.11	0.963	0.910	0.961	0.941	0.964	0.971
adaBoost	95.87	0.989	0.978	0.961	0.957	0.959	0.976
**Proposed Model**	**98.20**	**0.991**	**0.994**	**0.982**	**0.971**	**0.990**	**0.981**

**Table 7:** Repeated five (05)-fold cross-validation results.

Fold	Iteration 1	Iteration 2	Iteration 3	Iteration 4	Iteration 5	
1	98.24	98.21	97.88	98.19	97.89	**Mean of (A)** **(overall accuracy)**
2	97.91	97.87	97.65	98.01	97.89	
3	98.11	98.15	98.76	97.34	97.90	
4	98.03	98.32	97.65	98.21	98.02	
5	97.71	98.30	97.96	97.89	97.62	
Mean Acc./iteration (**A**)	**98.00**	**98.17**	**97.98**	**97.93**	**97.86**	**97.99**

**Table 8:** Validation results of the proposed ensemble model and its comparison with existing techniques.

SARS-CoV-2 Peptide Sequences	Actual Class	Binding Capacity by NetMHC	Predictions by CTLpred	Predictions by the Proposed Model
APAICHD	1	37	1	1
TAPAICHD	1	58	1	1
QLNRALTGIAVEQDK	1	6.2	-	1
NFSQILPDPSKPSKR	1	3.1	-	1
DILSRLD	1	65	1	1
TGSNVFQTR	1	45	1	1
HSSGVTREL	1	23	1	1
YICGFIQQK	1	4.2	1	1
VVCTEIDPK	1	8.2	1	1
TIWFLLLSV	1	76	1	1
TIADYNYKL	1	9.8	1	1
SYYSLLMPI	1	65	1	1
SVKGLQPSV	1	12	1	1
SQDLSVVSKT	1	19	-	1
QLEMELTPV	1	42	1	1
QLEMELTPV	1	7.3	1	1
NYNYRYRLF	1	1.9	1	1
NIADYNYKL	1	44	1	1
LLIIMRTFK	1	71	1	1
KLDGFMGRI	1	6.0	1	1
HTITVEELK	0	4.6	0	0
SVKHVYQL	0	52	0	0
EYHLMSFPQSAPHGV	0	79	-	0
DIKNLSKSL	0	80	0	0
VWNLDY	0	40	0	0
VTLAILTAL	0	32	0	0
YLNTLTLAV	0	41.2	0	0
EPVLKGVKL	0	5.6	0	0
AAGLEAPFL	0	9.3	0	0
WTAGAAAYY	0	4.4	0	0
YLDGADVTK	0	83	0	0
SQLGGLHLL	0	65	0	0
LVKPSFYVY	0	12	0	0
LPYPDPSRI	0	15.7	0	0
AEWFLAYIL	0	4.4	0	0
VLLSVLQQL	0	11	0	0
SLPSYAAFATA	0	89	-	0
TLMNVLTLV	0	37	0	0
IPLTTAAKL	0	61	0	0

## Data Availability

Publicly available datasets were analyzed in this study. This data can be found here: https://www.iedb.org/ (accessed on 2 August 2021).
